# Importation risks and local transmission of Japanese encephalitis virus in Taiwan

**DOI:** 10.1016/j.onehlt.2026.101422

**Published:** 2026-04-21

**Authors:** Yi-Chin Fan, Jo-Mei Chen, Chia-Hsin Lin, Yi-Ying Chen, Jia-Syuan Lin, Yuan-Dun Ke, Yao-Tsun Li, Shyan-Song Chiou

**Affiliations:** a Institute of Epidemiology and Preventive Medicine, College of Public Health, National Taiwan University, No. 17, Xuzhou Rd., Taipei 100025, Taiwan; b Master of Public Health Program, College of Public Health, National Taiwan University, No. 17, Xuzhou Rd., Taipei 100025, Taiwan; c Graduate Institute of Microbiology and Public Health, College of Veterinary Medicine, National Chung Hsing University, No. 145, Xingda Rd., Taichung 402202, Taiwan; d School of Biodiversity, One Health & Veterinary Medicine, University of Glasgow, 464 Bearsden Rd., Glasgow G61 1QH, United Kingdom

**Keywords:** Japanese encephalitis virus, Importation, Local transmission, Genetic evolution

## Abstract

Japanese encephalitis (JE) remains a significant threat to the human, horse, and pig industries throughout the Asia–Pacific region. Emerging genotype V and IV JE virus (JEV) can evade vaccine-induced immunity and exacerbate the disease burden after spreading into JE-naïve or epidemic areas through the movement of mosquitoes, pigs, and birds. This study aimed to assess the risk of viral importation into Taiwan by conducting mosquito-based surveillance. Our phylogenetic results revealed that at least six JEV importation events occurred in Taiwan between 1958 and 2024. Over the past 16 years of mosquito-based surveillance, three imported events of two genotype I (GI) subclusters (Sub I and Sub II) were detected and sustained local transmission in Taiwan from the first identification in 2008 and 2009 to 2024, without evidence of repeated importations. We further identified three distinct evolutionary stages of local GI JEVs: (1) an initial decade (2009–2018) characterized by co-circulation of Sub I and Sub II; (2) a reduction in viral population size, a decline in genetic heterogeneity, and the extinction of Sub II after 2018; and (3) emergence of a novel, dominant Sub I-I cluster bearing the *E*-V441I substitution in the stem region of envelope (E) protein. Our findings suggest that Taiwan has faced a low risk of JEV importation over the past 67 years. However, when the virus was introduced, it could subsequently establish a long-term circulation and overwinter in Taiwan. These findings underscore the importance of ongoing surveillance of potential JEV overwintering hosts to guide future control strategies.

## Highlights

• Mosquito-based surveillance revealed a low frequency of JEV importation into Taiwan.
• Genotype I JEV has maintained local transmission and overwintering for 17 years.
• A distinct, locally evolved subcluster I-I of JEV was identified in Taiwan.

## 1 Introduction

Japanese encephalitis virus (JEV) is a primary etiological agent of acute encephalitis in Asia and Australia [1], [2]. The virus is maintained in an enzootic transmission cycle primarily involving pigs and *Ardeidae* birds, with *Culex tritaeniorhynchus* serving as the principal vector. Pig farms surrounded by paddy fields provide an ideal environment for JEV circulation, as these settings serve as feeding and breeding habitats for both *Culex tritaeniorhynchus* and *Ardeidae* birds. JEV can occasionally spill over to dead-end hosts, such as humans and horses, causing encephalitis but not contributing to further transmission. The estimated global incidence of Japanese encephalitis (JE) is approximately 100,000 cases per year, with case-fatality rates ranging from 5% to 50%. Among survivors, an average of 56% experience long-term neurological sequelae [2], [3]. JE imposes a significant public health burden, resulting in an estimated annual loss of 709,000 disability-adjusted life years (DALYs) and prolonged economic strain on healthcare systems, which is expected to last at least a decade [4], [5]. Human vaccination is an effective strategy for reducing JE incidence [3]; however, breakthrough infection can still occur [4], and vaccination alone cannot interrupt JEV transmission within its zoonotic cycle, allowing the virus to persistently circulate in endemic and epidemic regions. Therefore, integrating a One Health approach into JEV surveillance and prevention is essential, including surveillance of mosquitoes, pigs, and birds, as well as measures to prevent animal–mosquito–animal and animal–mosquito–human transmission. Mosquito vectors play the most significant role in bridging the cross-species transmission of JEV, although the potential for vector-free transmission was reported [6].

JEV is a single-stranded, positive-sense RNA virus with an ∼11-kilobase genome that encodes a single polyprotein, which is subsequently cleaved into three structural proteins and seven non-structural proteins [2]. Based on the envelope (E) gene, JEV is classified into five genotypes (GI–GV) [7], each exhibiting distinct geographical distributions influenced by climatic factors [8]. Since the 1990s, GI has gradually replaced genotype III (GIII) as the dominant circulating genotype in subtropical and temperate regions of Asia [9]. In contrast, genotypes II (GII) and GIII remain prevalent in tropical countries such as Indonesia and the Philippines, respectively [10]. GIV was reported in an outbreak in Australia in 2022 [1], while GV expanded into South Korea in 2010, eventually replacing GI as the sole circulating genotype after 2020. This genotype shift was accompanied by a fourfold increase in JE cases in South Korea, as compared to the periods 2001–2009 and 2020–2024 [[11] Infectious Disease Statistics from Korea Disease Control and Prevention Agency; https://npt.kdca.go.kr/pot/is/inftnsdsEDW.do]. The replacement of GI by GV has raised concerns regarding the efficacy of current GIII-derived JE vaccines [12], [13], [14], particularly in regions experiencing seasonal JE and genotype shifts [9], [10].

Seasonal JEV strains may be introduced from endemic areas by viremic migratory birds or may persist locally through overwintering [15]. Phylogeographic analyses have demonstrated the long-distance dissemination of GI and its subclusters [16], [17], [18]. Notably, the migratory routes of birds overlap with observed viral expansion pathways, suggesting a potential role in virus dispersal [19]. However, certain seasonal JEV strains exhibit close phylogenetic relationships with past isolates from the same region, indicating local overwintering [8], [11], [15]. The local overwintering mechanisms for arbovirus had been proposed, including persistence in invertebrate hosts (such as mosquitoes and ticks) via horizontal or vertical transmission and maintenance in vertebrate reservoirs (such as pigs, *Ardeidae* birds, bats, and snakes), either through latent infection or vector-free transmission [6], [20]. Countries with seasonal JE outbreaks, such as China, Japan, and South Korea, have reported both overwintering and newly introduced viral strains, depending on the timing and location of surveillance [9], [10], [16], [17], [21]. Effective public health interventions should therefore incorporate viral surveillance in migratory birds and potential overwintering hosts, in addition to conventional vector control and vaccination strategies targeting humans and sows [14]. However, the specific mechanisms and hosts involved in JEV overwintering remain poorly understood, and a suitable research setting with continuous and local overwintering has yet to be identified.

In Taiwan, seasonal JEV activity peaks in mosquitoes and humans between May and July [22]. GIII JEV was the sole circulating genotype in Taiwan before 2008. In 2008 and 2009, the GIb clade I (subcluster II, Sub II) and clade II (subcluster I, Sub I) were first identified [23], [24]. Subsequently, a genotype shift from GIII to GI occurred in 2011–2012 [22]. Phylogeographic analyses of GI E genes and complete coding regions suggest that Taiwan GI JEVs originated from China and South Korea [9], [17], [18]. This study aims to investigate whether GI JEV has established a local transmission cycle or is frequently imported from other countries, and to elucidate the viral colonization process in a novel environment by analyzing a 17-year collection of mosquito-derived viral sequences from Taiwan. Our findings will provide insights into whether Taiwan serves as an ideal location for identifying potential overwintering hosts of JEV.

## 2 Materials and methods

### 2.1 Study design and mosquito sample collection

We conducted mosquito surveillance for JEV in Taiwan from 2009 to 2024, excluding 2013, 2014, and 2016. Each year, mosquitoes were captured from pig farms in eastern Taiwan (Yilan and Hualien), central Taiwan (Taichung, Changhua, and Yunlin), or southern Taiwan (Chiayi and Tainan) using UV-light traps from 5 pm to 9 pm from May to July. Female mosquitoes of the same species were pooled, homogenized, and centrifuged to collect the supernatant. The supernatant was subsequently stored at −80 °C until use.

### 2.2 Viral gene detection and sequencing

Viral RNA was extracted from the supernatant using the Viral RNA Extraction Miniprep System (VIOGENE-BioTek, New Taipei City, Taiwan). The extracted RNA was reverse-transcribed into complementary DNA (cDNA) using SuperScript III Reverse Transcriptase (Thermo Fisher Scientific, Waltham, MA, USA). JEV genes were then detected via multiplex RT-PCR with primers listed in Supplementary Table S1 [25]. Samples testing positive for JEV were further amplified by PCR and sequenced for the viral envelope (E) gene using primers listed in Supplementary Table S1. The virus E sequences identified in this study were uploaded to NCBI and listed in Supplementary Table S2.

### 2.3 Maximum likelihood tree of JEV sequences

All available JEV E genes were downloaded from the NCBI on July 13, 2025. We only included field isolates with full-length E sequences (1500 base) and a known collection year and country. Sequences were aligned by MAFFT [26] and edited by MEGA12 [27]. We excluded the recombinant JEVs detected by RDP5 v5.61 software [28] and removed the duplicate sequences collected from the same country in the same year, month, or date. The resulting alignments of 1597 JEV E genes were used to select the substitution model (Tamura and Nei [TN] substitution model with a gamma distribution and invariant sites) and construct a maximum likelihood tree using IQ-tree [29]. We further built a maximum likelihood tree using 966 GI JEV E genes by the same methods.

### 2.4 Time-scaled phylogenetic analysis of GI JEV

The temporal signals of 1597 GI to GV and 966 GI JEV E sequences were assessed by importing the maximum likelihood tree into TempEst [30]. The regressions of root-to-tip divergence were fit. The 1597 JEV E genes presented genotype-specific divergence and were challenging to converge in Bayesian inference. Ultimately, our dataset included 962 GI JEV E sequences (removed four outlier sequences) for time-scaled phylogenetic analysis. A time-scaled phylogenetic tree was inferred by using BEAST X v10.5.0 [31] and the BEAGLE 4 library [32]. We used the General Time Reversible (GTR) substitution model with a gamma distribution, the uncorrelated relaxed molecular clock with a lognormal distribution, and a Skygrid as a coalescent prior for the tree. This analysis was run for 2.7 billion Markov chain Monte Carlo (MCMC) steps, with samples collected every 10,000 iterations. The first 10% of stages were discarded as burn-in. We assessed the convergence and mixing results using all parameters with an effective sample size (ESS) value of ≥200, as determined by Tracer 1.7.2. [33] and generated a Maximum clade credibility tree by using TreeAnnotator 10.5.0 [34].

### 2.5 Skygrid reconstruction

The time-scaled phylogenetic inference for 158 GI, 118 Sub I, or 40 Sub II E sequences was conducted by using BEAST X v10.5.0 [31] and BEAGLE 4 library [32]. We used the TN substitution model with gamma distribution and invariant sites, the uncorrelated relax molecular clock with a lognormal distribution, and Bayesian Skygrid as tree prior with estimation of five grid points per year. We ran this analysis for 100 or 10 million MCMC chains, with samples collected every 10,000 or 1000 iterations, respectively. The first 10% of sampling trees were discarded as burn-in. We assessed the convergence and mixing results with all parameters ≥200 of ESS values and generated a Skygrid plot by using Tracer 1.7.2 [33]. The evolutionary rate of Sub I and Sub II was retrieved from the log file.

### 2.6 Genetic diversity and signatures of GI JEV isolates from Taiwan

The overall mean diversity of the E genes among Sub I and Sub II was estimated using the TN model with a gamma distribution, implemented in MEGA12 software [27]. To ensure balanced representation, Sub I and Sub II isolates were resampled to equalize the number of isolates from the same city in the same year. Isolates were excluded from the analysis if only one subcluster was identified in a given city per year. The genetic diversity for all resampling datasets of Sub I or Sub II isolates was averaged and reported as the overall mean genetic distance, along with its standard deviation (nucleotide substitutions per site).

A total of 118 Sub I isolates (collected between 2009 and 2024) and 40 Sub II isolates (collected between 2008 and 2018) were analyzed for genetic signatures using MEGA12 software [27]. Genetic signatures were identified if they were present in at least two of the most recent isolates. The temporal changes in the frequency of each genetic signature were visualized using GraphPad Prism version 8.0.2.

### 2.7 Adaptive selection analysis

We analyzed positive selection at specific branches and sites among Sub I and Sub II isolates from Taiwan using the adaptive Branch-Site Random Effects Likelihood (aBSREL) and Mixed Effects Model of Evolution (MEME) algorithms in Datamonkey (https://www.datamonkey.org/) [35].

### 2.8 Data availability

All alignment sequences and phylogeny results were deposited in Figshare under the “JEV sequence analysis” project name (https://doi.org/10.6084/m9.figshare.30373039).

### 2.9 Ethical considerations

No approval was required to collect mosquitoes and use available NCBI sequences.

## 3 Results

### 3.1 The risk of imported JEVs for Taiwan

We assessed how frequently Taiwan has faced the risk of imported JEVs. Since 2009, we have conducted continuous JEV surveillance using mosquitoes captured from pig farms across nine cities in Taiwan (Supplementary Table S3). Between 2009 and 2024, our surveillance identified 123 GI JEV E gene sequences, with no evidence of other co-circulating genotypes.

**Fig. 1:**
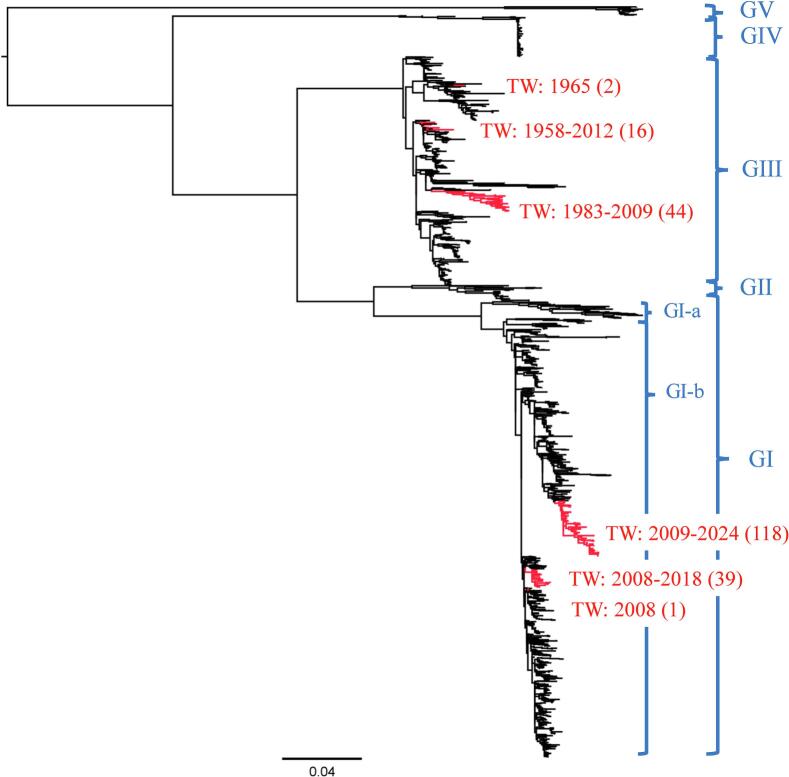
Maximum-likelihood phylogenetic tree of JEV isolates from Taiwan and other countries. All JEV isolates were classified into five genotypes: I, II, III, IV, and V (GI, GII, GIII, GIV, and GV). Genotype I viruses were further divided into GI-a and GI-b. Isolates originating from Taiwan (TW) are highlighted in red. The year of collection is indicated for each phylogenetically related group of Taiwanese isolates, and the number of isolates is shown in parentheses. The scale bar represents the number of nucleotide substitutions per site. (For interpretation of the references to colour in this figure legend, the reader is referred to the web version of this article.)

We analyzed the phylogeny of these 123 GI isolates, together with all other available JEV sequences from Taiwan and other Asia–Pacific countries, collected between 1939 and 2024 (Fig. 1 and Supplementary Fig. S1). The majority of JEV E sequences were derived from China, Japan, and Taiwan, followed by Australia, India, and South Korea (Supplementary Fig. S1a). Compared with GII, GIV, and GV, GIII and GI viruses were widely distributed and co-circulated in multiple countries from 2000 to 2020 (Supplementary Fig. S1b-f).

Our phylogenetic analyses revealed that at least six imported JEV events occurred in the past 67 years in Taiwan (Fig. 1). At least three imported GIII subclusters had been detected and circulated before 2012 (Fig. 1). The dominant GIII subcluster formed a monophyletic group and was maintained locally from 1983 to 2009 (ultrafast bootstrap support values ≥95%). Similarly, two GI subclusters were detected in Taiwan on three separate occasions, and two imported GI viruses subsequently formed two monophyletic groups that established long-term local transmission after 2008 (ultrafast bootstrap support values ≥95%) (Fig. 1). Together, these results indicate that Taiwan has experienced at least six independent JEV introduction events over the past 67 years, suggesting a relatively low but recurrent risk of viral importation. However, once introduced, imported JEVs were capable of sustaining local transmission for extended periods.

### 3.2 First importation and local transmission of GI JEVs in Taiwan

**Fig. 2:**
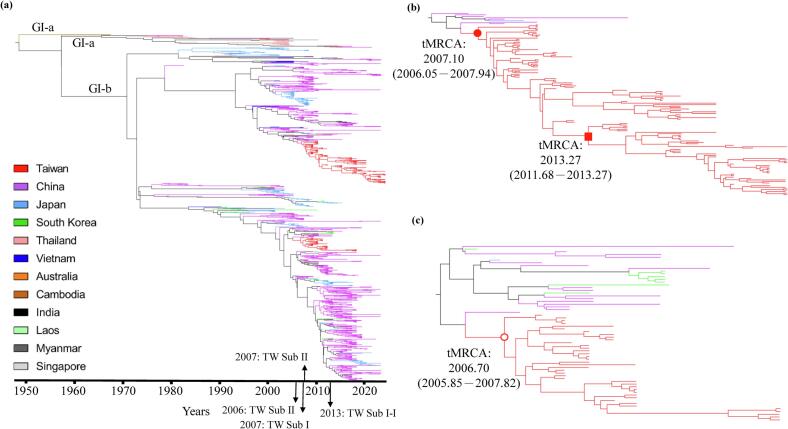
Maximum clade credibility (MCC) tree of genotype I (GI) JEVs from Taiwan and other countries. (a) The MCC tree includes 962 GI isolates from 12 countries, each represented by a distinct colour. The time scale (in years) is shown below the tree. The inferred introduction years of Taiwan Sub I and Sub II were indicated, and the emergence of Sub I-I was additionally labeled. (b, c) Enlarged phylogenies of Taiwan Sub I and Sub II isolates, respectively, both supported by high posterior probabilities (posterior = 1). Sub I has further evolved into Sub I-I, also with a high posterior probability (posterior = 1). The estimated times to the most recent common ancestor (tMRCA) for Taiwan Sub I (filled red circle), Sub II (filled red square), and Sub I-I (open red circle) are indicated as median years, with the 95% highest posterior density (HPD) intervals shown in parentheses. (For interpretation of the references to colour in this figure legend, the reader is referred to the web version of this article.)

Two GI subclusters were first detected in Taiwan in 2008 and 2009, but their exact introduction times remained uncertain. Using Bayesian phylogenetic inference (Fig. 2a), we estimated that Sub I and Sub II viruses were introduced at a similar time in the fall and winter of 2006, approximately 2 to 3 years before their first detection (Fig. 2b and c). Following their introductions, two subclusters were transmitted within Taiwan and persisted for the subsequent years, with the longest lasting until 2024 (Fig. 2 and Supplementary Fig. S1b). In contrast, GI isolates from China, Japan, South Korea, India, Cambodia, Vietnam, Laos, Thailand, and Myanmar formed distinct phylogenetic clusters separate from the Taiwanese isolates (Fig. 2a and Supplementary Fig. S1b).

Phylogenetic and phylogeographic analyses indicated that China, Japan, and South Korea were the most likely sources of the initial GI introductions into Taiwan (Fig. 2b and c) [17], [34]. However, their contemporaneous GI isolates between 2001 and 2023 were phylogenetically distinct from the Taiwanese strains, and in South Korea, GI viruses were entirely replaced by GV after 2014 (Fig. 2 and Supplementary Fig. S1f). Collectively, these results provide strong evidence that GI JEV has established a local transmission cycle in Taiwan and has overwintered locally without evidence of reintroduction from other countries since 2009.

### 3.3 Temporal dynamics of sub I and sub II viruses, along with genetic adaptation

**Fig. 3:**
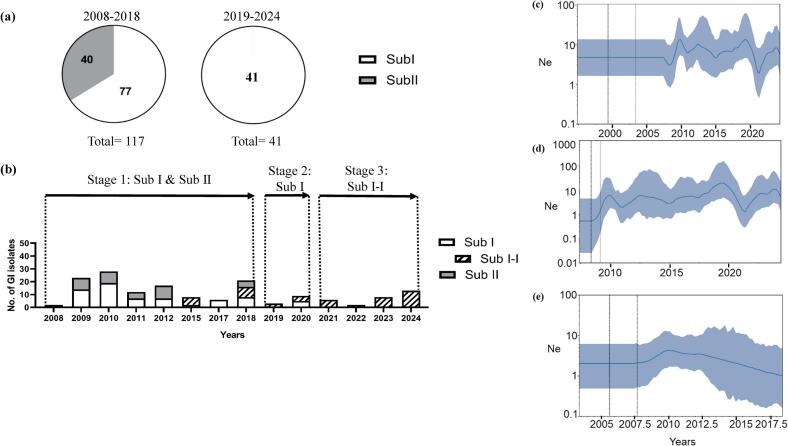
Temporal dynamics of GI Sub I and Sub II JEVs in Taiwan, 2008–2024. (a) Pie charts illustrate the number of Sub I (white area) and Sub II (gray area) isolates identified during two time periods: 2008–2018 and 2019–2024. (b) The annual number of Sub I, Sub I-I, and Sub II isolates is represented by empty bars, empty bars with diagonal stripes, and filled bars, respectively. The arrows and dotted lines illustrate the time spans of three stages (Stage 1, Stage 2, and Stage 3) of GI virus adaptation. (c-e) Population dynamics of all GI (c), Sub I (d), and Sub II (e) JEVs were inferred by using the Skygrid model. The posterior median of viral effective population size (Ne) with the 95% Bayesian credibility interval was presented as the solid line and shaded regions. The best estimate for the time of the tree root and the upper highest posterior density were indicated with the vertical lines.

Taiwan GI JEVs exhibited a three-stage (Stage I, Stage II, and Stage III) adaptive process over their 17-year local transmission (Fig. 3). Sub I and Sub II co-circulated for the first decade (2009–2018) across central, southern, and eastern pig farms, except in 2015 and 2017, when only one pig farm was sampled (Fig. 3a and b). During this period, Sub I was more prevalent, with 77 isolates compared to 40 Sub II isolates (Fig. 3a). Following 2018, Sub II disappeared. Its proportion was significantly decreased from 34.19% (40/117) during 2008 to 2018 to 0% (0/41) during 2019 to 2024 (one-sided Fisher's exact test, *p* < 0.05). Sub I became the dominant subcluster in the second stage (Fig. 3b). The final stage has been characterized by the dominance of a distinct subcluster within Sub I, Sub I-I, which was first detected in 2015 and originated in the spring of 2013 (Figs. 2b and 3b). The Sub I-I has subsequently become the dominant strain since 2021 (Fig. 3b). Taiwan Sub I JEVs took approximately 7 years to evolve into a new subcluster (Sub I-I) after their first arrival in the winter of 2006 (Fig. 2b). The change-point analysis of the proportions of Sub I, Sub I-I, and Sub II further supported the transitions from Stage I to Stage II and from Stage II to Stage III (Supplementary Fig. S2).

The similar dynamic pattern of Sub I and Sub II isolates was reflected in the virus population dynamics (Fig. 2c-e). All GI and Sub I JEVs showed a relatively stable population. Still, Sub II exhibited a declining population and a decreased genetic diversity after 2010 (Supplementary Fig. S3). However, Sub I (1·1904 × 10^−3^ subs/site/year; 95% HPD: 8·804 × 10^−4^ to 1·4961 × 10^−3^) and Sub II (1·4402 × 10^−3^ subs/site/year; 95% HPD: 8·7677 × 10^−4^ to 2·1029 × 10^−3^) were estimated with comparable evolutionary rate.

**Fig. 4:**
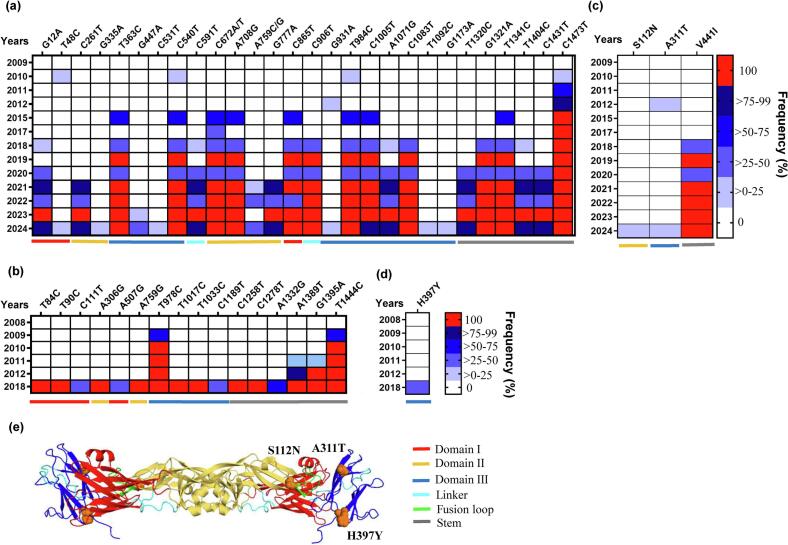
Temporal frequency and structural location of GI Sub I and Sub II mutations in the viral envelope protein. (a–d) The earliest available Sub I isolate from 2009 and Sub II isolate from 2008 in Taiwan were used as reference sequences to identify nucleotide (a and b) and amino acid (c and d) mutations in the envelope (E) protein. Only mutations detected in ≥2 isolates from 2024 are presented. The annual frequency of mutations was analyzed in Sub I (a and c) and Sub II (b and d) isolates. (e) SWISS-MODEL (https://swissmodel.expasy.org/) was used to simulate the E dimers of JEV GI YL2009–4 virus with a PDB template of 3P54. The structural locations of the Sub I and Sub II amino acid substitutions within Domain I (red), Domain II (yellow), Domain III (blue), the linker region (cyan), and fusion loop (green) of the E protein are shown. The stem region (gray) was unresolved in the crystal structure of E protein. (For interpretation of the references to colour in this figure legend, the reader is referred to the web version of this article.)

We analyzed substitutions and evolutionary selection that occurred in Sub I and Sub II through three stages of adaptive process (Fig. 4). From 2008 to 2017, only synonymous mutations were detected (Fig. 4); however, in 2018, a V441I substitution in the stem region of E protein emerged in 50% (8/16) of Sub I isolates (Fig. 4c and e), while an H397Y substitution in Domain III was observed in 40% (2/5) of Sub II isolates (Fig. 4d and e). The Sub I-I isolate first detected in 2015, carrying silent mutations in the E gene (Fig. 4a). Some Sub I-I isolates acquired the V441I substitution in 2018; by 2021, all Sub I-I isolates carried this mutation (Fig. 4c). In 2024, minor Sub I-I isolates further evolved the S112N substitution in Domain II or the A311T substitution in Domain III (Fig. 4c and e). The evolutionary analyses indicated no evidence of episodic diversifying selection in Sub I. They assessed S112N, V441I, H397Y, and A311T substitutions as neutral mutations. The viral factors associated with the extinction of Sub II and the dominance of Sub I-I in Taiwan remain to be elucidated.

## 4 Discussion

Emerging GIV and GV JEVs have shown reduced sensitivity to vaccine-induced antibodies and can spread over long distances through mosquito dispersal, pig transportation, and migratory birds [16], [19]. In subtropical and temperate regions of Asia, seasonal JE outbreaks are often irregularly driven by imported viruses and/or locally overwintering strains [9], [10], [16], [21], [36]. Using phylogenetic inference, we estimated that JEV importation into Taiwan occurred at a low frequency—approximately six independent events over the past 67 years, including three GI introductions during the past 17 years. Notably, two of the three imported GI viruses [66.7% (2/3)] successfully established subsequent long-term local transmission (Fig. 1, Fig. 2). Our phylogenetic analyses further revealed that GI JEV has continuously circulated and overwintered in Taiwan for 17 years without new viral introductions. This prolonged endemic circulation allowed us to observe viral adaptation and competition between two co-circulating GI subclusters, and the evolution of distinct Sub I-I isolates. In the aspect of long-term and local virus circulation and overwintering, this study demonstrated that Taiwan is an ideal place to elucidate overwinter hosts for JEV, which could serve as an alternative target for virus surveillance and prevention.

Compared to China, Japan, and Korea, which have frequently introduced GI JEV to each other or from southeastern countries, Taiwan has experienced only three introductions from China or Korea in 2008 and 2009 [9], [18], [36]. Despite China, our primary spatial source of JEV, exhibiting high GI virus activity under intensive surveillance efforts from 2008 to 2021 (Supplementary Fig. S1) [17], all GI isolates collected from 2008 to 2024 in Taiwan were phylogenetically related to each other rather than China isolates (Fig. 2). In addition to Taiwan, Okinawa and South Korea may serve as suitable locations for studying overwintering mechanisms of JEV. All available GI isolates from Okinawa, an island geographically close to Taiwan, collected in 1998, 2004, and 2008, clustered closely in the phylogenetic tree (Supplementary Fig. S4), suggesting local viral transmission. However, recent surveillance has failed to detect JEV in mosquitoes captured at U.S. military installations in Okinawa, and no human JE cases were reported between 2016 and 2021 [37]. Since 2010, GV has been exclusively circulating in South Korea, suggesting the potential for localized transmission (Supplementary Fig. S1) [38].

The pig–mosquito–pig transmission cycle is considered the most critical for maintaining JEV in endemic regions and during the JE season in epidemic regions [20]. In countries with seasonal JE epidemics, viral transmission from amplifying hosts to overwintering host(s), and vice versa, may further facilitate viral persistence during the winter non-epidemic season and subsequently contribute to JE outbreaks in the following year. These transmission processes are most likely mosquito-mediated, as vector-free transmission of JEV appears insufficient to sustain viral circulation [6]. Therefore, year-round pig production and the continuous presence of *Culex* mosquitoes in Taiwan may support long-term JEV transmission and overwintering. Migratory birds arrive in Taiwan during April to May and October to January, both of which occur after the onset of the JE season. This pattern may be associated with our observation of a low frequency of JEV introduction, thereby reducing the potential confounding effects of imported viruses when investigating local overwintering hosts. JEV-positive mosquitoes were detected earliest and persisted longest in southern Taiwan, suggesting that this region may be particularly suitable for investigating overwintering hosts. Future investigations of overwintering hosts could focus on resident *Ardeidae* birds, bats, and mosquitoes inhabiting wetland and woodland ecosystems, caves, underground areas, and urban rooftops in regions with the earliest onset and longest duration of the JE season, with sampling conducted at the end of the non-epidemic season or approximately two months before the start of the JE epidemic season.

We observed dynamic fluctuations in the number of co-circulating Sub I and Sub II isolates during the first decade, followed by the exclusive circulation of Sub I and the subsequent emergence of Sub I-I in Taiwan. Previous analysis of the effective population size also indicated a decline in Sub II (GIb Clade I) activity and the expansion of Sub I (GIb Clade II) after 2016 in Asia [17]. The similar exponential growth of an emerging subclade within Sub I (GIb Clade II) was reported in China [36], mirroring our observations of Sub I-I. However, the signature substitutions in Taiwan Sub I-I differ from those of the emerging Chinese subclade, which encodes NS2a-151 V and NS4b-20 K substitutions [17]. Instead, our locally circulating GI strains exhibit unique E protein substitutions, including *E*-V441I, E-H397Y, and/or E-A311T, providing strong evidence for local viral adaptation in Taiwan. The 40% of Sub II isolates carried the E-H397Y substitution, which may influence interactions between E dimers or affect the conformational rearrangement of the E protein during pH change [39]. The currently dominant Sub I-I strains retain *E*-V441I and/or E-A311T substitutions. Although E-V441I was considered a neutral selection, its *p*-value of 0.194 is close to significance (*p* ≤ 0.1) for diversifying selection. The E-441 was located on the stem region of the E protein and was involved in the stability of the flavivirus postfusion structure [40]. Interestingly, the E-A311T substitution identified in two Sub I-I isolates in 2024 was also present in a Taiwanese GI strain from 2012. The recently emerging GV isolates encode a serine residue at position 311 of the E protein, distinct from other genotypes [12], [13]. This S311 substitution has been associated with reduced neutralizing activity against GV in individuals immunized with GIII-based JE vaccines [14]. Further experimental studies are needed to determine whether the A311T mutation affects the neutralizing activity of antibody responses elicited by current GIII-derived vaccines.

Mosquitoes play a central role in cross-host JEV transmission during the epidemic season and overwinter [20]. Based on 157 JEV E gene sequences collected over 17 years through long-term mosquito surveillance, our study provides compelling phylogenetic and genetic evidence for the local transmission of GI JEV in Taiwan. These findings highlight the feasibility and importance of mosquito-based surveillance for assessing the risks of JEV importation and local transmission, and support surveillance of potential overwintering hosts as an important component of JEV control and prevention. The identification of the unique Sub I-I subcluster and the E-A311T substitution further emphasizes the importance of continuous JEV surveillance in both endemic and epidemic regions, particularly in light of potential GV virus evasion [14]. A limitation of this study is the lack of complete genome sequences for all isolates. Future studies will aim to identify potential locations of overwintering hosts by conducting phylogeographic analyses of JEV dispersal patterns using the full coding sequences from a broader range of geographic locations in Taiwan.

## Authors contribution

Conceptualization, Y.F. and S.C.; methodology, Y.F. and Y.L.; formal analysis, Y.F.; investigation, J.C., C.L., Y.C., J.L, and Y.K.; resources, S.C. and Y.F.; data curation, Y.F., C.L., and J.C.; writing—original draft preparation, Y.F.; writing—review and editing, S.C.; visualization, Y.F.; supervision, Y.F. and S.C.; funding acquisition, S.C. and Y.F. All authors have read and agreed to the published version of the manuscript.

## CRediT authorship contribution statement

**Yi-Chin Fan:** Writing – original draft, Supervision, Resources, Methodology, Funding acquisition, Formal analysis, Data curation, Conceptualization. **Jo-Mei Chen:** Investigation, Data curation. **Chia-Hsin Lin:** Investigation, Data curation. **Yi-Ying Chen:** Investigation. **Jia-Syuan Lin:** Investigation. **Yuan-Dun Ke:** Investigation. **Yao-Tsun Li:** Methodology. **Shyan-Song Chiou:** Writing – review & editing, Supervision, Resources, Funding acquisition, Conceptualization.

## Declaration of generative AI and AI-assisted technologies in the writing process

We utilized ChatGPT and Grammarly to edit our English and enhance readability. After using the tools, we reviewed and revised as needed.

## Funding sources

This study was supported by the National Science and Technology Council (grand number NSTC 114–2314-B-005-002-MY3, 113–2314-B-002-169-MY3, MOST 110–2313-B-005-044, and 110–2314-B-005-003-MY3); the 10.13039/501100002701Ministry of Education in Taiwan (grant number NTU-114L9004).

## Declaration of competing interest

All authors declare no competing interests.

## Data Availability

I have shared the linked to my data in the materials and methods.

## Appendix A Supplementary data

Supplementary materials and data Image 1
