# Supplementary material for: Importation risks and local transmission of Japanese encephalitis virus in Taiwan
Source: One Health. 2026 Apr 21;22:101422. doi: 10.1016/j.onehlt.2026.101422 (PMC13129449; doi:10.1016/j.onehlt.2026.101422)
Supplement: Supplementary file 1 — Supplementary materials and data [file mmc1.docx]

**Supplementary Table S1.** Primers used for JEV multiplex RT-PCR (mRT-PCR), PCR, and sequencing^a^.

| Primers | Sequence (5’ to 3’) | Methods |
| --- | --- | --- |
| UniJEV F | TGTGTGAACTTCTTGGCTTAGTAT | mRT-PCR |
| UniJEV R | CARCATCTGTTYTCWCCTTTTGA | mRT-PCR |
| GIII-specific primer F | GGATGCTTGGCAGTAACAAC | mRT-PCR |
| GIII-specific primer R | AAGTCCACATCCGTTGCC | mRT-PCR |
| GI-specific primer F | CAGTCGCGAGTTTAAACGAC | mRT-PCR |
| GI-specific primer R | CATTCAGTTCGTCCCGCACA | mRT-PCR, PCR, and sequencing |
| GIJEV 931-951F | CGTGTGGTATTCACTATTCTC | PCR |
| GIJEV 1587-1607R | GACTTCGAACCCACGGTCATG | sequencing |
| GIJEV 1456-1475F | GGAGCGTCTCAAGCAGCAAA | sequencing |
| GIJEV 2102-2120R | GAGTCTCCGAAGGGAGGTT | sequencing |
| GIJEV 2000-2019F | CAGTCGCGAGTTTAAACGAC | sequencing |

^a^JEV, Japanese encephalitis virus; RT-PCR, reverse transcription

**Supplementary Table S2**. Information on genotype I Japanese encephalitis viruses collected in this study

| Strains | Collection year | Accession no. |
| --- | --- | --- |
| TC2009-1 | 2009 | JF499791 |
| TC2009-2 | 2009 | JF499792 |
| TC2009-5 | 2009 | JF499795 |
| TC2009-6 | 2009 | JF499796 |
| TC2009-7 | 2009 | JF499797 |
| TC2009-8 | 2009 | JF499798 |
| TC2009-9 | 2009 | JF499799 |
| TC2009-11 | 2009 | JF499801 |
| TC2009-12 | 2009 | JF499802 |
| TC2009-14 | 2009 | JF499804 |
| HL2010-1 | 2010 | JF499827 |
| TC2010-1 | 2010 | JF499810 |
| TC2010-2 | 2010 | JF499811 |
| TC2010-3 | 2010 | JF499812 |
| TC2010-4 | 2010 | JF499813 |
| TC2010-6 | 2010 | JF499815 |
| YL2010-3 | 2010 | JF499818 |
| CH2010-1 | 2010 | JF499819 |
| CH2010-2 | 2010 | JF499820 |
| CH2010-3 | 2010 | JF499821 |
| TN2010-1 | 2010 | JF499826 |
| TC2011-1 | 2011 | PV405869 |
| TC2011-5 | 2011 | PV405870 |
| TC2015-1 | 2015 | PV405871 |
| TC2015-4 | 2015 | PV405872 |
| TC2015-5 | 2015 | PV405873 |
| TC2015-6 | 2015 | PV405874 |
| TC2015-7 | 2015 | PV405875 |
| TC2015-8 | 2015 | PV405876 |
| TC2015-9 | 2015 | PV405877 |
| TC2015-10 | 2015 | PV405878 |
| TC2017-1 | 2017 | MZ733965 |
| TC2017-2 | 2017 | MZ733966 |
| TC2017-3 | 2017 | MZ733967 |
| TC2017-4 | 2017 | MZ733968 |
| TC2017-5 | 2017 | MZ733969 |
| TC2017-6 | 2017 | MZ733970 |
| YiL2018-1 | 2018 | PV405885 |
| YiL2018-2 | 2018 | PV405886 |
| YiL2018-3 | 2018 | PV405887 |
| YiL2018-4 | 2018 | PV405888 |
| YiL2018-6 | 2018 | PV405889 |
| YiL2018-7 | 2018 | PV405890 |
| YiL2018-8 | 2018 | PV405891 |
| YiL2018-11 | 2018 | PV405892 |
| YL2018-1 | 2018 | PV405895 |
| YL2018-2 | 2018 | PV405896 |
| YL2018-3 | 2018 | PV405897 |
| YL2018-4 | 2018 | PV405898 |
| YL2018-5 | 2018 | PV405899 |
| YL2018-6 | 2018 | PV405900 |
| TC2018-4 | 2018 | MT075627 |
| TC2018-5 | 2018 | MT075628 |
| TC2019-1 | 2019 | MT075629 |
| TC2019-2 | 2019 | MT075630 |
| TC2019-3 | 2019 | MT075631 |
| TC2020-1 | 2020 | PV405904 |
| TC2020-2 | 2020 | PV405905 |
| TC2020-3 | 2020 | PV405906 |
| TC2020-4 | 2020 | PV405907 |
| TC2020-5 | 2020 | PV405908 |
| TC2020-6 | 2020 | PV405909 |
| YL2020-1 | 2020 | PV405910 |
| YL2020-2 | 2020 | PV405911 |
| YL2020-3 | 2020 | PV405912 |
| TC2021-1 | 2021 | PV405913 |
| TC2021-2 | 2021 | PV405914 |
| TC2021-3 | 2021 | PV405915 |
| TC2021-4 | 2021 | PV405916 |
| TC2021-5 | 2021 | PV405917 |
| TC2021-6 | 2021 | PV405918 |
| TC2022-1 | 2022 | PV405919 |
| TC2023-1 | 2023 | PV405920 |
| TC2023-2 | 2023 | PV405921 |
| TC2023-3 | 2023 | PV405922 |
| TC2023-4 | 2023 | PV405923 |
| TC2023-5 | 2023 | PV405924 |
| TC2023-6 | 2023 | PV405925 |
| TC2023-7 | 2023 | PV405926 |
| TC2023-8 | 2023 | PV405927 |
| TC2024-1 | 2024 | PV405929 |
| TC2024-2 | 2024 | PV405930 |
| TC2024-3 | 2024 | PV405931 |
| TC2024-4 | 2024 | PV405932 |
| TC2024-5 | 2024 | PV405933 |
| TC2024-6 | 2024 | PV405934 |
| TC2024-7 | 2024 | PV405935 |
| TN2024-1 | 2024 | PV405936 |
| TN2024-2 | 2024 | PV405937 |
| TN2024-3 | 2024 | PV405938 |
| TN2024-4 | 2024 | PV405939 |
| TN2024-5 | 2024 | PV405940 |
| YiL2024-1 | 2024 | PV405928 |
| TC2009-3 | 2009 | JF499793 |
| TC2009-4 | 2009 | JF499794 |
| TC2009-10 | 2009 | JF499800 |
| TC2009-13 | 2009 | JF499803 |
| YL2009-1 | 2009 | JF499805 |
| YL2009-2 | 2009 | JF499806 |
| YL2009-3 | 2009 | JF499807 |
| YL2009-4 | 2009 | JF499808 |
| YL2009-5 | 2009 | JF499809 |
| TC2010-5 | 2010 | JF499814 |
| YL2010-1 | 2010 | JF499816 |
| YL2010-2 | 2010 | JF499817 |
| CY2010-1 | 2010 | JF499822 |
| CY2010-2 | 2010 | JF499823 |
| CY2010-3 | 2010 | JF499824 |
| CY2010-4 | 2010 | JF499825 |
| TC2011-2 | 2011 | PV405855 |
| TC2011-3 | 2011 | PV405856 |
| TC2011-4 | 2011 | PV405857 |
| TC2012-1 | 2012 | PV405858 |
| TC2012-2 | 2012 | PV405859 |
| TC2012-3 | 2012 | PV405860 |
| TC2012-4 | 2012 | PV405861 |
| TC2012-6 | 2012 | PV405863 |
| TC2012-7 | 2012 | PV405862 |
| YiL2018-5 | 2018 | PV405864 |
| YiL2018-9 | 2018 | PV405865 |
| TC2018-1 | 2018 | MT075624 |
| TC2018-2 | 2018 | MT075625 |
| TC2018-3 | 2018 | MT075626 |

**Supplementary Table S3**. **Genotype I Japanese encephalitis virus isolates identified in Taiwan, 2008 to 2024.**

| Years | No.^a^ | North | Central | | | South | | | East | | References |
| --- | --- | --- | --- | --- | --- | --- | --- | --- | --- | --- | --- |
|  |  | Taipei | Taichung | Changhua | Yunlin | Chiayi | Tainan | Kaohsiung | Yilan | Hualien |  |
| 2008 | 2 | 1 | 0 | - | 0 | - | 0 | 0 | 1 | 0 | 22 |
| 2009 | 23 | 2 | 16 | - | 5 | - | - | - | 0 | 0 | 20 &21& This study |
| 2010 | 28 | 3 | 9 | 3 | 3 | 4 | 1 | - | 1 | 4 | 20 &21& This study |
| 2011 | 14 | -^c^ | 11 | - | - | - | - | - | 3 | - | 20 & This study |
| 2012 | 18 | 2 | 9 | - | - | - | 3 | 1 | 2 | 1 | 20 & This study |
| 2015 | 9 | - | 9 | - | - | - | - | - | - | - | This study |
| 2017 | 6 | - | 6 | - | - | - | - | - | - | - | This study |
| 2018 | 21 | - | 5 | - | 6 | - | - | - | 10 | - | This study |
| 2019 | 3 | - | 3 | - | - | - | - | - | - | - | This study |
| 2020 | 9 | - | 6 | - | 3 | - | - | - | - | - | This study |
| 2021 | 6 | - | 6 | - | - | - | - | - | - | - | This study |
| 2022 | 2^b^ | - | 1 | - | - | - | - | - | - | - | NCBI&This study |
| 2023 | 8 | - | 8 | - | - | - | - | - | - | - | This study |
| 2024 | 13 | - | 7 | - | 0 | - | 5 | - | 1 | - | This study |

^a^ No., number

^b^ One isolate without isolation location

^c^ “-” indicates not available.


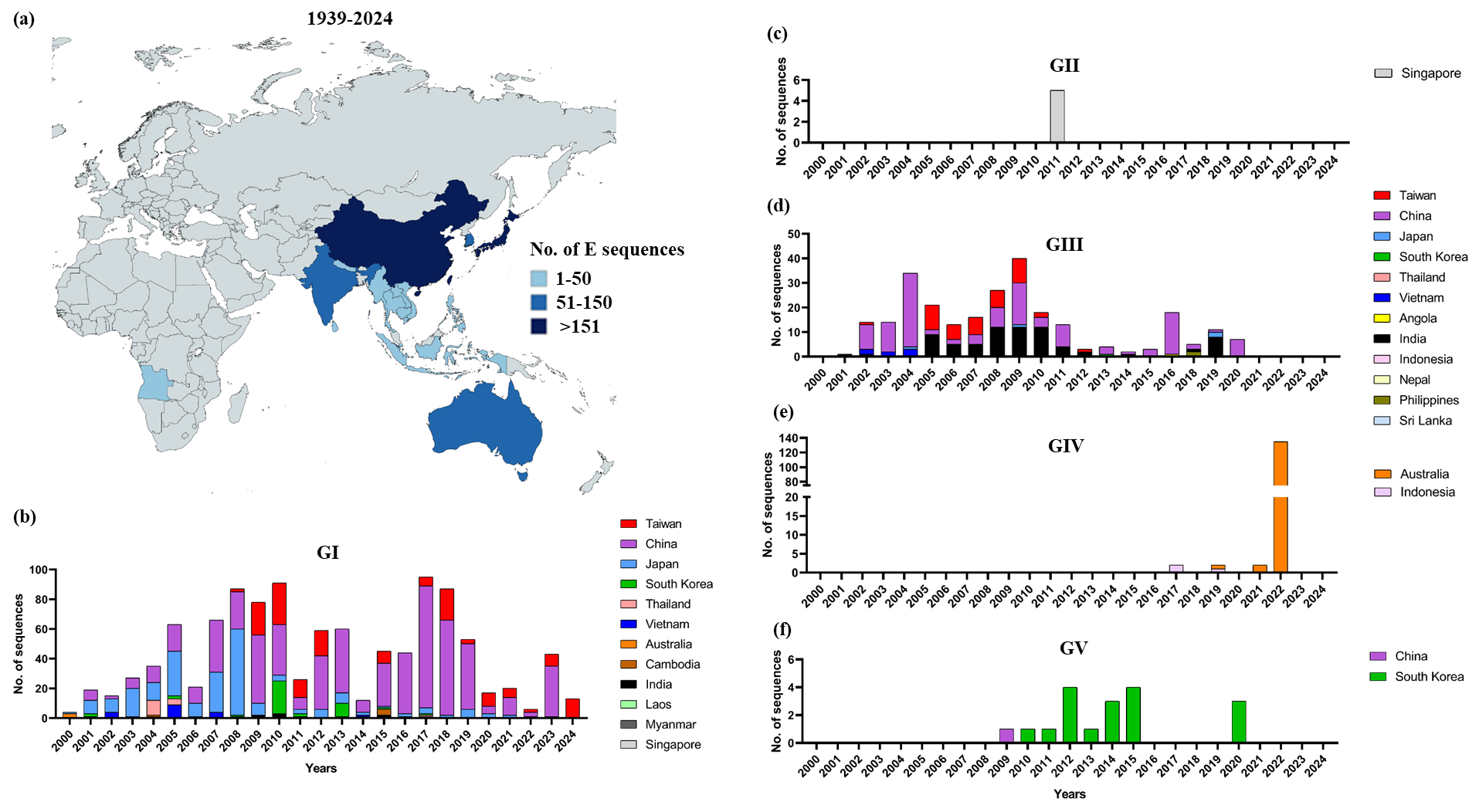


**Supplementary Fig. S1. Geographical and temporal distribution of JEV envelope (E) gene sequences representing five genotypes.** (a) All available complete E gene sequences collected from 1935 to 2024, including those obtained in this study and from the NCBI database. (b–f) Annual numbers of genotype I–V (GI, GII, GIII, GIV, and GV) E gene sequences detected in different countries after 2000.


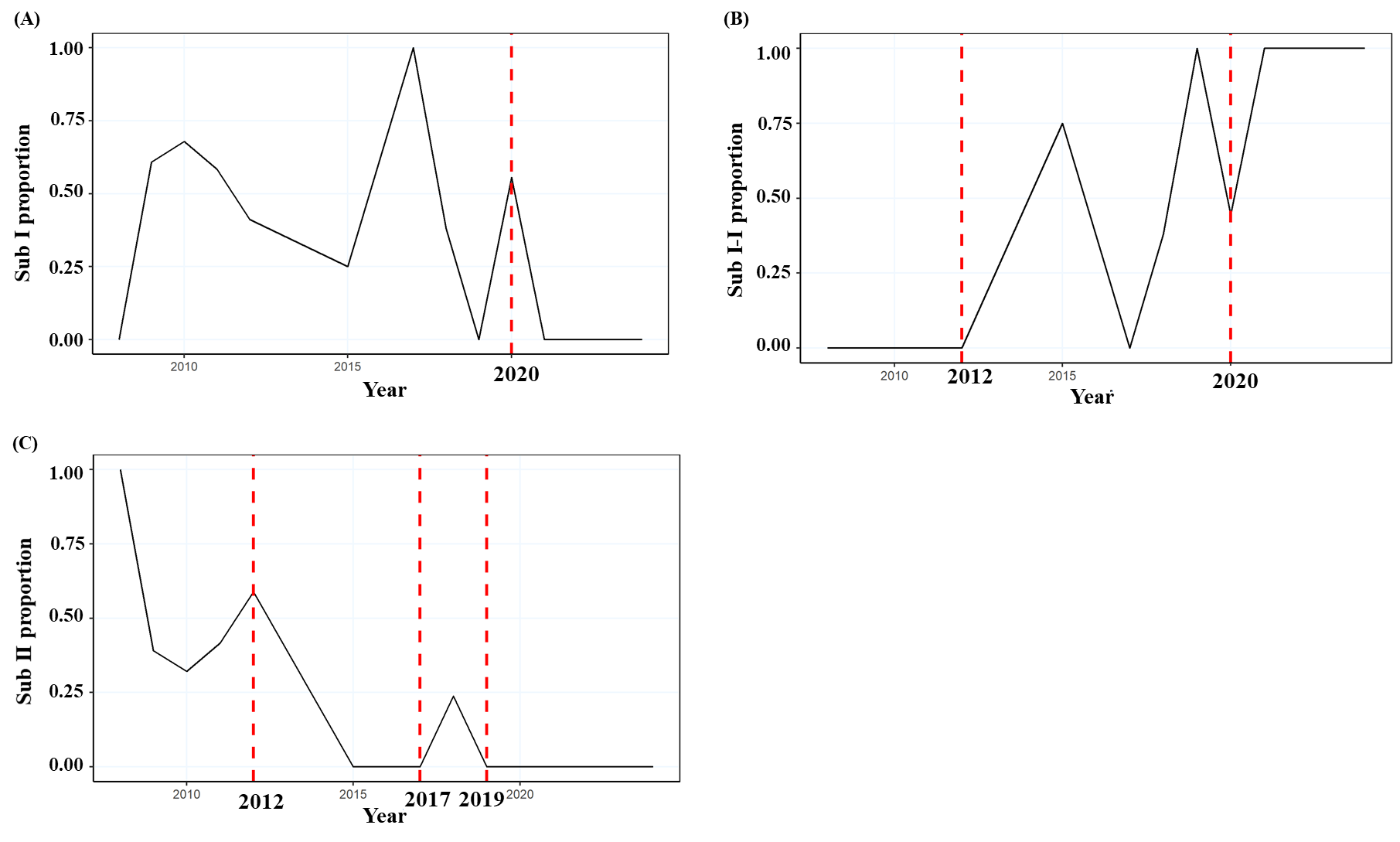


**Supplementary Fig. S2. The change-point analysis of the proportions of Sub I (A), Sub I-I (B), and Sub II (C) during 2008-2024.** The change point (Year) was indicated by the red dotted lines. The R package changepoint was used in this analysis.


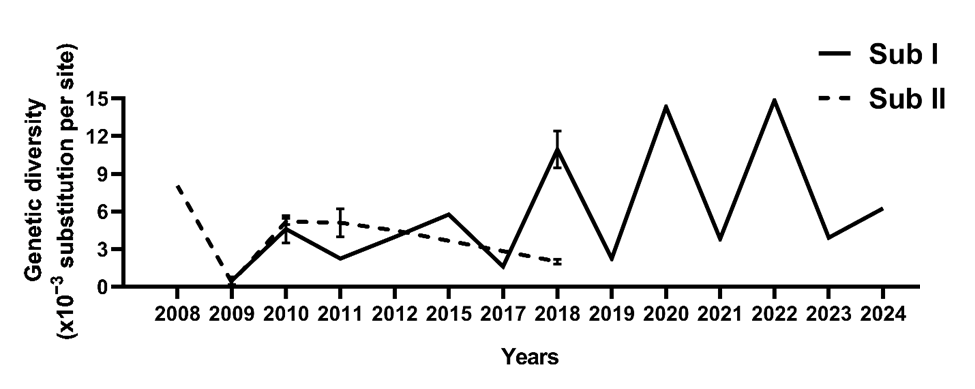


**Supplementary Fig. S3. Genetic diversity of GI Sub I and Sub II JEVs.** The temporal dynamics of mean genetic diversity ± standard deviation for the envelope protein of Sub I and Sub II isolates are shown as a solid line and a dashed line, respectively. To ensure comparability, the Sub I and Sub II isolates included in the analysis were resampled to equalize the sample sizes of the two subclusters from the same city.


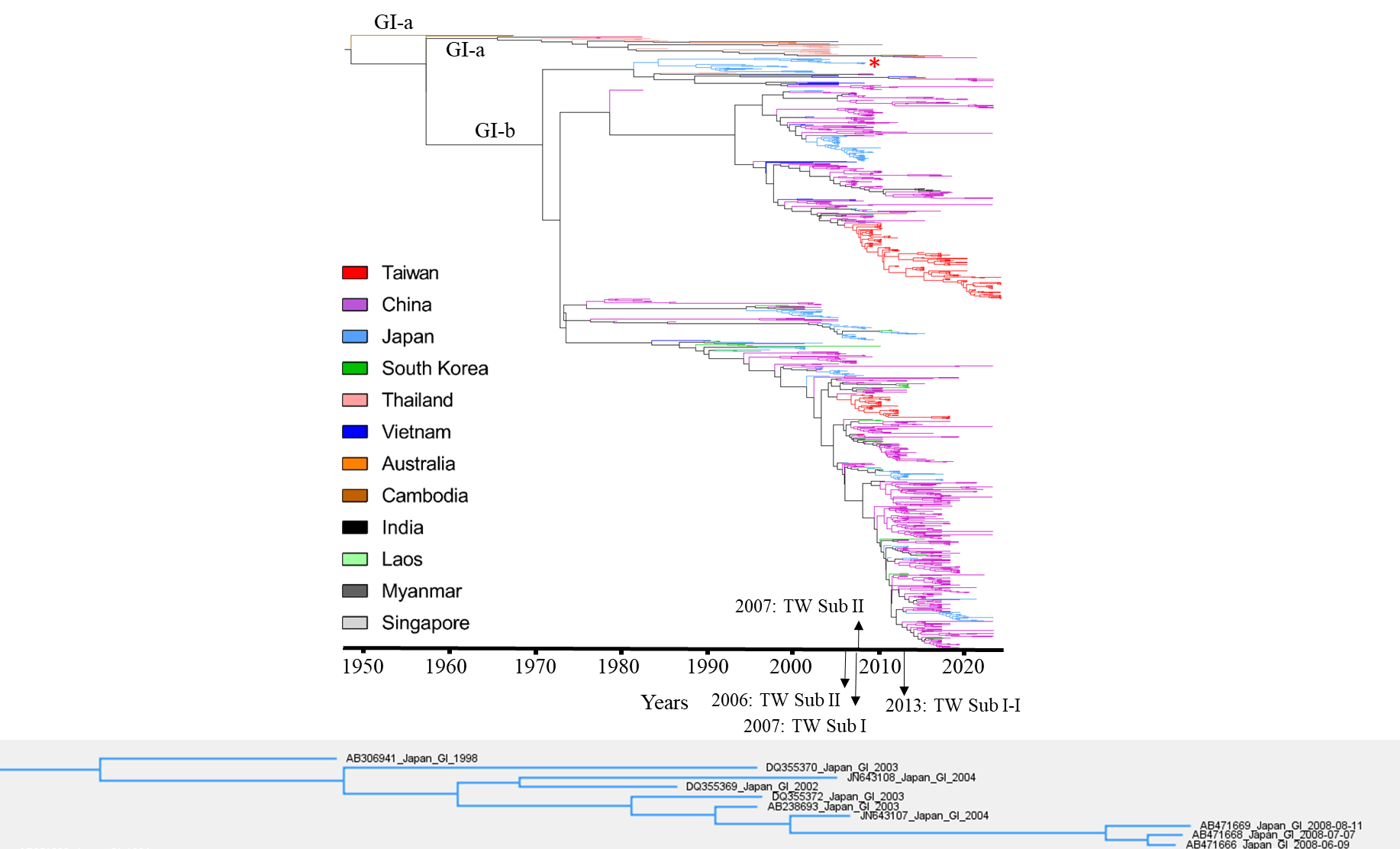


**Supplementary Fig. S4. Phylogenetic relation of GI isolates from Okinawa, Japan.** The MCC tree was retrieved from Fig. 2a. A red asterisk indicates the branches of Okinawa GI isolates, as shown below.
